# Characterization of an *AGAMOUS* gene expressed throughout development of the fleshy fruit-like structure produced by *Ginkgo biloba* around its seeds

**DOI:** 10.1186/s12862-015-0418-x

**Published:** 2015-07-16

**Authors:** Alessandro Lovisetto, Barbara Baldan, Anna Pavanello, Giorgio Casadoro

**Affiliations:** Department of Biology, University of Padua, Via G. Colombo, 3, 35131 Padua, Italy; Botanical Gardens, University of Padua, Via Orto Botanico, 15, 35123 Padua, Italy

**Keywords:** *AGAMOUS* gene, Fleshy fruit-like structures, *Ginkgo biloba*, MADS-box genes, *Solanum lycopersicon*

## Abstract

**Background:**

The involvement of MADS-box genes of the *AGAMOUS* lineage in the formation of both flowers and fruits has been studied in detail in Angiosperms. *AGAMOUS* genes are expressed also in the reproductive structures of Gymnosperms, yet the demonstration of their role has been problematic because Gymnosperms are woody plants difficult to manipulate for physiological and genetic studies. Recently, it was shown that in the gymnosperm *Ginkgo biloba* an *AGAMOUS* gene was expressed throughout development and ripening of the fleshy fruit-like structures produced by this species around its seeds. Such fleshy structures are evolutionarily very important because they favor the dispersal of seeds through endozoochory. In this work a characterization of the Ginkgo gene was carried out by over-expressing it in tomato.

**Results:**

In tomato plants ectopically expressing the Ginkgo *AGAMOUS* gene a macroscopic anomaly was observed only in the flower sepals. While the wild type sepals had a leaf-like appearance, the transgenic ones appeared connately adjoined at their proximal extremity and, concomitant with the development and ripening of the fruit, they became thicker and acquired a yellowish-orange color, thus indicating that they had undergone a homeotic transformation into carpel-like structures. Molecular analyses of several genes associated with either the control of ripening or the ripening syndrome in tomato fruits confirmed that the transgenic sepals behaved like ectopic fruits that could undergo some ripening, although the red color typical of the ripe tomato fruit was never achieved.

**Conclusions:**

The ectopic expression of the Ginkgo *AGAMOUS* gene in tomato caused the homeotic transformation of the transgenic sepals into carpel-like structures, and this showed that the gymnosperm gene has a genuine C function. In parallel with the ripening of fruits the related transgenic sepals became fleshy fruit-like structures that also underwent some ripening and such a result indicates that this C function gene might be involved, together with other gens, also in the development of the Ginkgo fruit-like structures. It seems thus strengthened the hypothesis that *AGAMOUS* MADS-box genes were recruited already in Gymnosperms for the development of the fleshy fruit habit which is evolutionarily so important for the dispersal of seeds.

**Electronic supplementary material:**

The online version of this article (doi:10.1186/s12862-015-0418-x) contains supplementary material, which is available to authorized users.

## Background

Members of the *AGAMOUS* (*AG*) lineage of MADS-box genes have been shown to perform important functions in the development of flowers and fruits in Angiosperms [1, 2]. *AG* genes are expressed also in the reproductive structures of Gymnosperms, and in some cases their function has been demonstrated by ectopically expressing them in the dry-fruit producing Arabidopsis. For instance, Tandre et al. [3] obtained the floral homeotic conversion that is expected for the *AG* genes by the over-expression of a *Picea* gene in Arabidopsis, while Zhang et al. [4] demonstrated the *AG* identity of a gene from *Cycas edentata* by using it to complement the *ag* loss-of-function mutant of Arabidopsis. Therefore, the *AG* genes appear to be involved in the specification of the reproductive structures in both Gymnosperms and Angiosperms (i.e., the Spermatophytes, or seed plants), also together with the B-function genes in the case of the male structures [5, 6].

In general, the situation of the *AGAMOUS* genes in Gymnosperms is less known compared to that of Angiosperms. A recent genomic work by Gramzow et al. [7], carried out using both sequenced genomes and sets of transcriptomes, evidenced the presence of more than one copy of *AG* genes in a few species. It is interesting to note that some *AG* genes were found to be expressed not only in the reproductive structures but also in various vegetative tissues [7]. In Angiosperm core eudicots two sub-lineages of *AGAMOUS* are usually found: the *euAGAMOUS* (*euAG*) and *PLENA* (*PLE*) ones [6], whose representative members can perform different functions according to the plant species ([8] and references therein). In particular, various studies have shown that *AGAMOUS* genes are important also for the formation of fruits, besides the long known role played by them in flowers. In tomato a detailed analysis of the functional roles played by each representative of the two *AG* sub-lineages (i.e., the *euAG TAG1* and the *PLE TAGL1* genes, respectively) demonstrated that both genes are involved in the early stages of fruit development, while it is *TAGL1* to be especially important for the process of ripening [8]. Accordingly, in tomato plants over-expressing the *euAG TAG1* gene the normally leafy sepals were transformed into fleshy structures that became yellowish-orange [9], while tomato plants over-expressing the *PLE*-like *TAGL1* gene produced fleshy sepals that accumulated lycopene and became red as ripe tomato fruits normally do [10]. Similarly red and fleshy sepals were obtained also by Tadiello et al. [11] in tomato plants over-expressing a *PLE*-like gene from peach. Interestingly, members of the *PLE* subgroup appeared to be involved in the process of ripening in both climacteric and non-climacteric fruits [8, 10–13].

From an evolutionary point of view, the appearance of seeds represented the turning point that allowed the Spermatophytes to radiate all over the world [14]. Seeds contain an embryo that can survive for a long time after being released by the plant, thus seeds represent a moment in which a whole plant can be mobile. Plants have developed various mechanisms to favor the dispersal of seeds, one of them consisting in equipping seeds with fleshy tissues attractive to frugivorous animals which would then disperse the seeds into the environment through their own excrements, a process known as endozoochory ([15] and references therein). So far, the molecular mechanisms involved in the formation of the fleshy tissues accompanying seeds have mostly been studied in Angiosperms because they can produce fleshy fruits of relevant economical importance like tomato, grape and others.

Also Gymnosperms can produce fleshy structures, and it was recently shown that an *AGAMOUS* gene was expressed throughout development and ripening of the fleshy fruit-like structures produced by two different gymnosperm species around their seeds: *Ginkgo biloba* and *Taxus baccata* [16]. Such a common characteristic appeared particularly interesting because the fleshy fruit-like structures of the two Gymnosperms had different anatomical origins. In *T. baccata* the fleshy aril was formed *de novo* starting as a ring at the base of the ovule, while in *G. biloba* it was the seed integument that developed into a fleshy fruit-like structure [16]. Accordingly, it was hypothesized that those two gymnosperm *AG* genes might have a common function in the development of the two different fruit-like structures [16].

In order to better understand the possible role played by the Gymnosperm *AGAMOUS* genes in the formation of fleshy fruit-like structures, a functional characterization of the *GBM5 AG* gene from Ginkgo was carried out in this work. However, being Ginkgo a woody species that is not amenable for transformation experiments, the gene was studied in tomato, a species that produces fleshy fruits and can be genetically engineered. Because of the heterologous system used in this work, only over-expression experiments were carried out for the characterization of the gene. The ectopic expression of the Ginkgo *AG* gene caused the homeotic transformation of the normally leafy tomato sepals into fleshy fruit-like structures that also underwent some ripening, as judged by their yellowish-orange color and by the expression of several ripening-related genes. These results show that the Ginkgo *AG* gene has a C function and is involved in the formation of fleshy fruit-like structures.

## Results

**Fig. 1:**
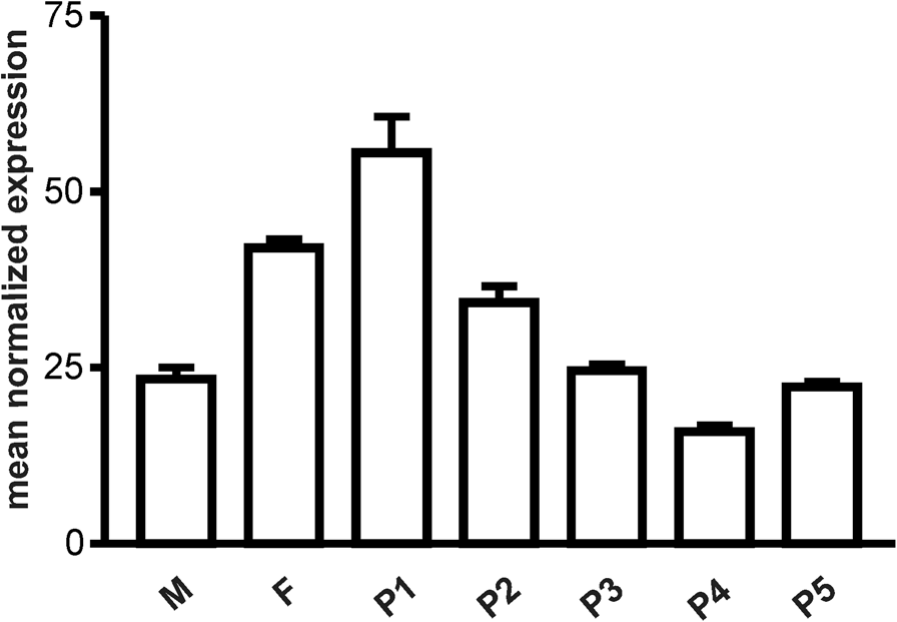
Relative expression profile of the *GBM5* gene in various Ginkgo tissues. M, male (stamens); F, female (very young ovules); P1, P2, P3, P4, P5, seed sarcotesta pulp at different stages of development (P1 to P4) and ripening (P5). Values (means of normalized expression) from real-time PCR analyses. Bars: standard deviations

The Ginkgo *AGAMOUS* gene (*GBM5*) studied in this work had already been described by Jager et al. [17], and its detailed expression in various tissues and during the development of the fleshy sarcotesta produced by this species around its seeds has recently been published by Lovisetto et al. [16]. Interestingly, this gene did not appear to be expressed in leaves but only in male and female reproductive structures. In situ hybridization analyzes evidenced that the *AG* gene was expressed already in very young ovules and its transcripts were present throughout the ovule [16] Analyses of GBM5 expression performed by real- time PCR showed the presence of transcripts in both male and female reproductive structures, particularly in young ovules (Fig. 1). As regards the fleshy fruit-like structures, GBM5 transcripts reached high levels during the early stage of fast growth (P1, Fig. 1), and then constantly decreased, although a further increase could be observed in samples at the ripening stage (P5, Fig. 1).

**Fig. 2:**
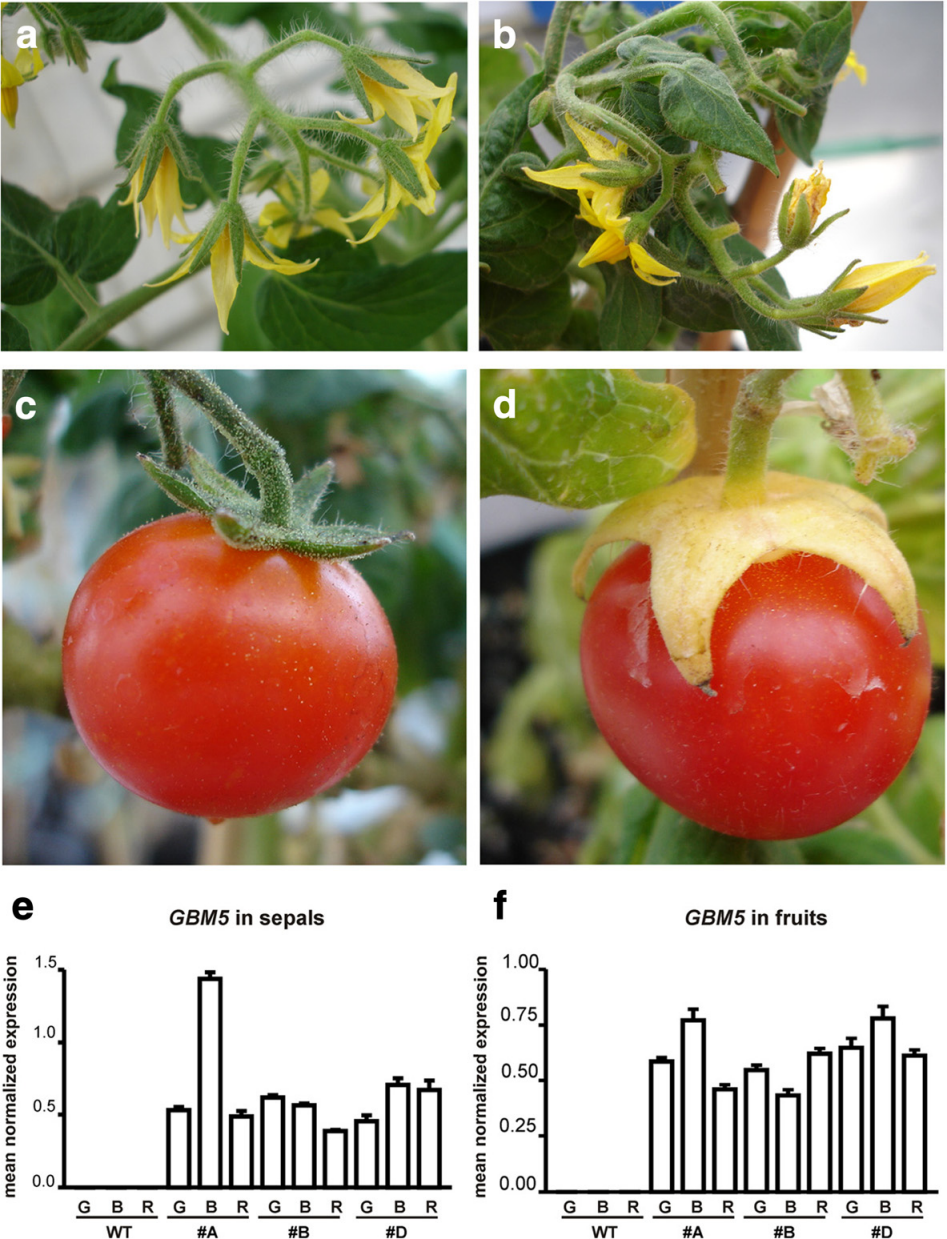
Phenotypic characterization of tomato flowers and fruits from wild type (**a**, **c**) and transgenic (**b**, **d**) #A plants, and analysis of the *GBM5* gene expression in sepals (**e**) and fruits (**f**) of both wild type and 35S:*GBM5* plants. Post-anthesis wild type (**a**) and transgenic #A (**b**) flowers look similar. Marked differences can be observed only in sepals accompanying the ripe fruit. Those of the wild type fruit (**c**) are green and have a leaf-like appearance while the corresponding sepals from the 35S:*GBM5* ripe fruit (**d**) appear thick, fused at the proximal extremity and with a yellowish-orange color. Panels E (sepals) and F (fruits) show the relative expression of the *GBM5* gene whose transcripts are found only in the samples obtained from the three different 35S:*GBM5* plants (#A, #B, #D) studied in this work. G, mature green fruits and their relative sepals; B, breaker fruits and their relative sepals; R, red fruits and their relative sepals. Values (means of normalized expression) from real-time PCR analyses. Bars: standard deviations

In this work the *GBM5* cDNA was used to generate transgenic tomato plants (cv Florida Petite) ectopically expressing it under the control of the strong constitutive 35S promoter [18]. Four transgenic lines were obtained that contained the transgene as verified by PCR analyses (not shown). In general, the vegetative apparatus did not show any apparent abnormality while three lines showed floral differences compared to wild types, all of them regarding the sole structure of the sepals. The actual expression of the Ginkgo *GBM5* gene was verified by real time PCR in sepals and fruits of the three lines and transgene transcripts were observed in all the transgenic samples, while no transcripts for this gene were detected in the wild type samples (Fig. 2e, f). The fruits of the three lines formed seeds so all the characterizations (i.e., morphological and molecular) were carried out with T2 generation plants.

### Phenotypic characterization of the tomato plants ectopically expressing the GBM5 gene

In wild type post-anthesis flowers the sepals were completely separated while in the transgenic flowers the sepals tended to remain fused at their proximal end (Fig. 2a, b). Subsequently, during growth and ripening of the berry the transgenic sepals became thicker, especially at the proximal end. On the contrary the wild type sepals remained separated and maintained a more leaf-like appearance. Concomitant with the proceeding of the berry ripening also the transgenic sepals underwent some ripening and their color changed from green to a yellowish-orange tonality while the fruit became red. In wild type plants the ripening of the berry was not accompanied by macroscopic changes of the sepals that remained green and maintained their leaf-like appearance (Fig. 2c, d for line #A and Additional file 1 for lines #B and #D).

**Fig. 3:**
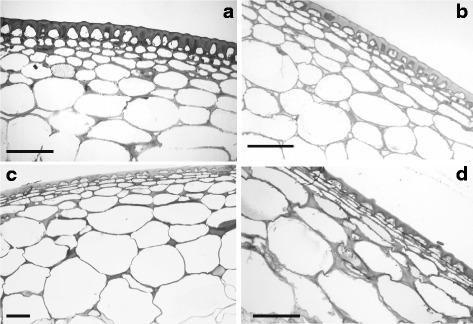
Light microscopy analysis of tomato fruits from wild type (**a**, **c**) and transgenic (**b**, **d**) plants. In the pericarp of mature green fruits of both wild type (**a**) and transgenic (**b**) plants it is possible to recognize an internal region consisting of large cells with small intercellular spaces, and an outer region formed by small cells with thickened walls situated underneath an epidermis with a thick layer of cuticle. In the red ripe fruits of both wild type (**c**) and transgenic (**d**) plants the above similar structure is maintained but for a marked lengthening of the cells in the outer pericarp region (scale bar = 100 μm)

**Fig. 4:**
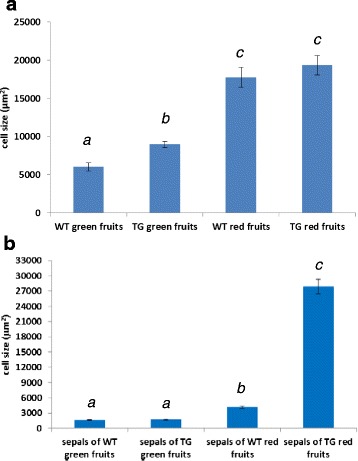
Cell area in wild type (WT) and transgenic (TG) fruits (**a**) and sepals (**b**) accompanying fruits at different stages of development. Values are means ± SE of 100 measured cell areas for each sample. Bars labeled with a different letter differ significantly (p < 0.05) by Student’s *t* test

The internal structure of pericarp and sepals from both wild type and transgenic plants was examined by light microscopy. In green fruits the internal part of the pericarp was formed by very large cells while the outermost part of the pericarp consisted of a few layers of small-sized cells whose walls had evident thickenings that gave them the appearance of collenchymas (Fig. 3a, b). During ripening the cells of the internal pericarp increased their size maintaining a somewhat roundish shape while the small cells of the external pericarp became lengthened with the longer side running parallel to the epidermis that, in all the examined fruits, appeared covered by a thick layer of cuticle (Fig. 3c, d). In general, no difference in pericarp thickness and structure was observed between transgenic (Fig. 3b, d) and wild type (Fig. 3a, c) fruits. In the internal pericarp the cells of transgenic green fruits had a slightly larger area compared to the corresponding wild type fruits although the difference did not appear to be statistically significant. As the fruit developed to the red stage of ripening the increase of cell size was sharp and their area doubled in respect to green fruits. Also in this case no significant differences were recorded in the cell size of transgenic red fruits compared to the corresponding wild type ones (Fig. 4a).

**Fig. 5:**
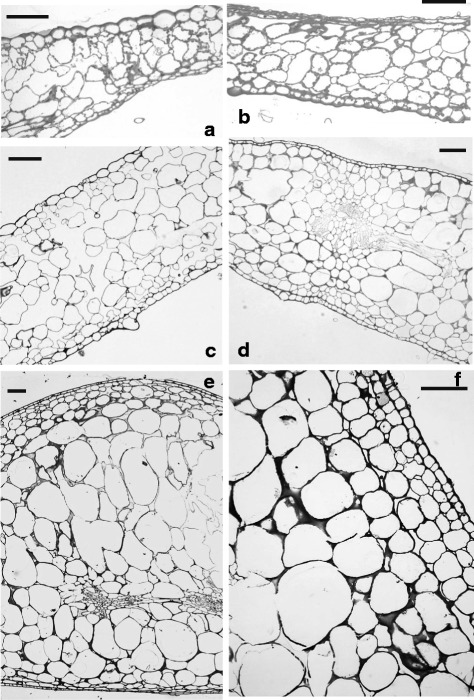
Light microscopy analysis of tomato sepals from wild type (**a**, **c**) and transgenic (**b**, **d**, **e**, **f**) plants. A leaf-like structure with a spongy mesophyll is visible in the wild type sepals accompanying both mature green (**a**) and red (**c**) fruits while in all the transgenic sepals (**b**, **d**, **e**, **f**) the internal structure appears rather compact. The thickness of the transgenic sepals show an increase in parallel with the passage from the mature green (**b**) to breaker (**d**) and red (**e**, **f**) stage of development of the fruits to which they are attached. A detail of a yellowish sepal (**f**) accompanying a red fruit shows that the small sized cells situated underneath the abaxial epidermis have a multilayered organization (scale bar = 100 μm)

In sepals the observed macroscopic differences between transgenic and wild type ones were accompanied also by internal anatomical differences. In general, wild type sepals maintained their leaf-like structure throughout development, although no palisade parenchyma was recognizable and the whole mesophyll appeared spongy (Fig. 5a, c). In parallel with the process of fruit ripening, the wild type sepals showed some slight increase in their thickness mostly due to enlargement of the mesophyll cells (Fig. 4b). The possibility that also an increased number of cell layers might contribute to the increased sepal thickness was investigated although the latter fact was difficult to be exactly assessed because the cells of the spongy parenchyma did not form regular tiers and were mixed with ample air spaces (Fig. 5c). Actually, a slight increase could be measured albeit it did not appear to be highly significant since the number of cell layers changed from about 6.6 (±0,2) to about 8.8 (±0,2) during the passage of fruits from the green to the red stage of development.

**Fig. 6:**
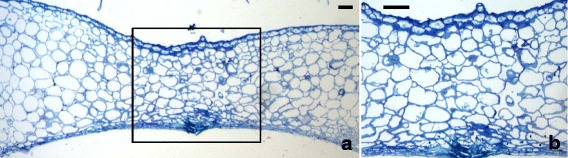
Light microscopy analysis of the zone where two yellowish transgenic sepals are fused together. In panel (**a**) a low magnification of the fused region is shown and no visible anatomic boundary can be detected where the two sepals join together (black rectangle). In panel (**b**) a higher magnification of the area marked by the black rectangle in (**a**) confirms the absence of anatomical boundaries. However, the mesophyll cells in the fusion zone are smaller compared to those located at the center of the sepal. (scale bar = 100 μm)

In transgenic plants the general anatomy of sepals accompanying the green fruits looked similar to the corresponding wild type ones, with a spongy mesophyll and no palisade tissue. Yet, the air spaces were smaller and the mesophyll appeared more compact than in wild type sepals (Fig. 5b). Concomitant with the ripening of fruits the transgenic sepals changed their structure and became much thicker, as it can be observed in Fig. 5d where a section of a sepal accompanying a fruit at the breaker stage is shown. The number of layers increased from about 7.6 (±0,4) to about 10 (±0,2) during the passage of fruits from the green to the red stage of development. In general the mesophyll appeared compact with small air spaces, therefore it was possible to see that the increased thickness was partly due to an increase in the number of cell layers, although it was an enlargement of the internal mesophyll cells that significantly contributed to the observed thickness increase (Fig. 4b). In particular, the process of thickening reached a maximum in the yellowish-orange sepals that accompanied the red ripe fruits (Fig. 5e, f). Measurements of cell area (Fig. 4) demonstrated that such marked sepal thickening was especially due to a huge cell enlargement. In sections through the region where two “ripe” transgenic sepals are fused together no visible anatomic boundary could be detected between the two sepals although the cells had a somewhat smaller size compared to those situated at the center of the sepals (Fig. 6).

But for the presence of two epidermises, the internal structure resembled that of the berry pericarp with an outer region consisting of small cells and an internal region filled with very large cells. The general internal structure of the “ripe” transgenic sepals appeared more compact and with small air spaces compared to that of the corresponding wild type sepals. In the area close to the abaxial epidermis the small-sized mesophyll cells were arranged to form a compact and layered region that showed up against the huge cells of the internal mesophyll (Fig. 5e, f).

### Molecular characterization of the tomato plants ectopically expressing the GBM5 gene

**Fig. 7:**
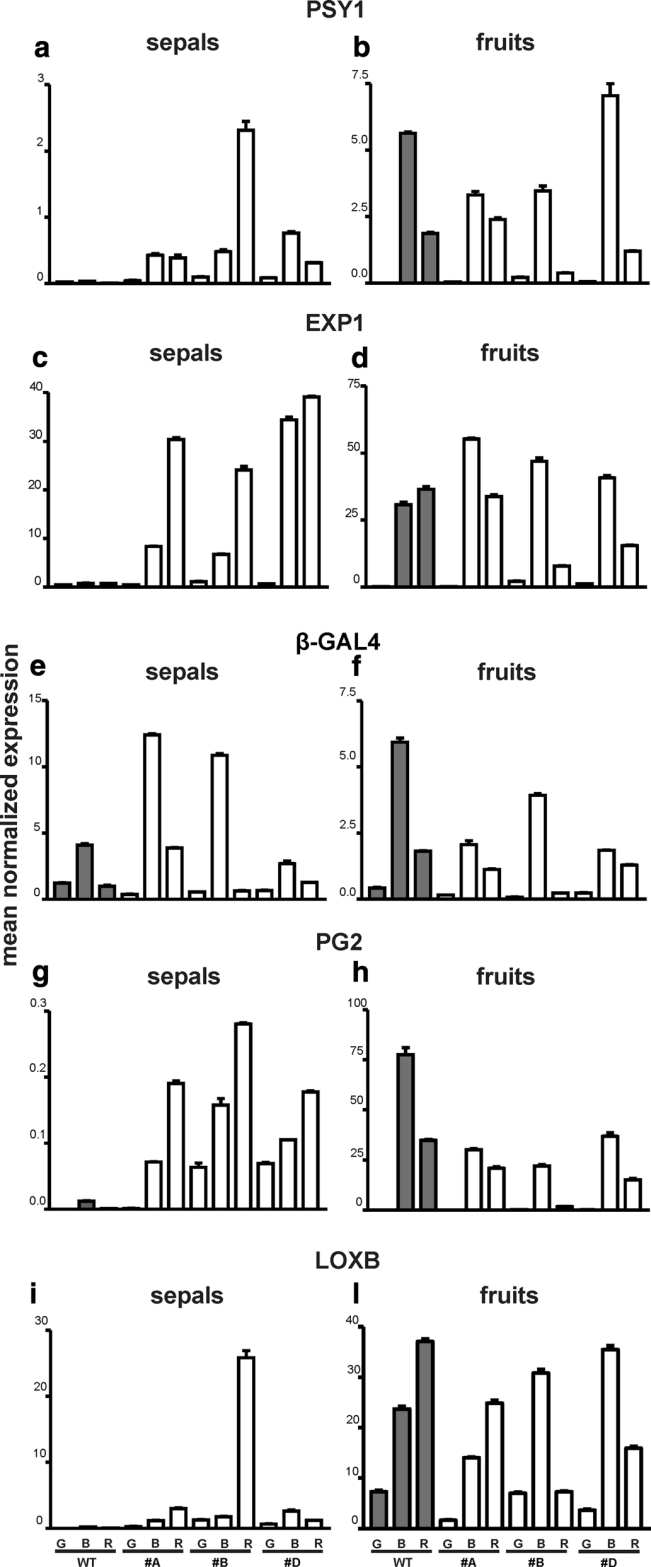
Relative expression profiles of genes related to ripening in tomato sepals and fruits of WT and 35S:*GBM5* plants. Grey rectangles represent wild type tissues (WT) while white rectangles represent 35S:*GBM5* tissues from three different lines (#A, #B, #D). G, mature green fruits and their relative sepals; B, breaker fruits and their relative sepals; R, red fruits and their relative sepals. The studied genes were *phytoene synthase1 (PSY1, panels* ***a*** *and* ***b*** *), expansin (EXP1, panels* ***c*** *and* ***d*** *), β-galactosidase (β-GAL4, panels* ***e*** *and* ***f*** *) polygalacturonase (PG2, panels* ***g*** *and* ***h*** *)* and *lipoxygenase (LOXB, panels* ***i*** *and* ***j*** *)*. Values (means of normalized expression) from real-time PCR analyses. Bars: standard deviations

During ripening, the tomato berry becomes red as the result of lycopene accumulation. Phytoene synthase 1 (*PSY1*) is the gene involved in the biosynthesis of carotenoids in ripening tomato fruits [19], therefore the expression of this gene was analyzed in both sepals and fruits during ripening. In wild type sepals the transcripts of this gene were almost undetectable, while in all the transgenic lines the *PSY1* gene showed increasing levels of expression in sepals concomitant with the acquisition of the yellowish-orange color. As regards the fruit, the *PSY1* gene showed an expression pattern that was comparable in both wild type and transgenic berries, with a maximum at the breaker stage when accumulation of carotenoids occurs (Fig. 7a, b).

During ripening fleshy fruits undergo a loss of firmness, a process known as softening, and several genes coding for different cell wall degrading enzymes are involved in this process [20, 21]. Three representatives of the above suite of genes were selected as markers of softening: expansin (*EXP1*), β-galactosidase (*β-GAL4*) and polygalacturonase (*PG2*). In fruits the expansin gene was similarly expressed in both wild type and transgenic samples (Fig. 7d), while in the case of sepals the gene transcripts were almost undetectable in wild type ones and showed a ripening-related increment in the transgenic sepals in parallel with the ripening of the fruit (Fig. 7c). Regarding *β-GAL4*, no relevant differences in the general pattern of expression were observed in either wild type or transgenic fruits during ripening, both of them showing a peak of gene transcripts at the breaker stage (Fig. 7f). In sepals there was some difference between the wild type ones and those of two transgenic lines, the latter having much higher expression levels especially in those that accompanied the fruits at the breaker stage (Fig. 7e). In the case of the *PG2* gene the pattern of expression was similar in all fruit types, albeit the wild type breaker fruits showed a higher expression level compared to the corresponding transgenic ones (Fig. 7h). Quite different was the situation in sepals where the gene transcripts were almost undetectable in wild type ones, and showed an increasing pattern of expression in the transgenic sepals in parallel with the ripening of the fruit (Fig. 7g).

Another aspect of ripening is the acquisition of particular flavors that make the ripe fruits desirable to animals. To this purpose, a lipoxygenase (*LOX* B) gene has been shown to be important for flavor formation in tomato [22, 23]. As expected, the expression of *LOX* B increased during ripening in both wild type and transgenic fruits (Fig. 7j). On the contrary, expression of the *LOXB* gene was almost undetectable in wild type sepals, while some expression of the gene could be observed in ripening transgenic sepals (Fig. 7i).

**Fig. 8:**
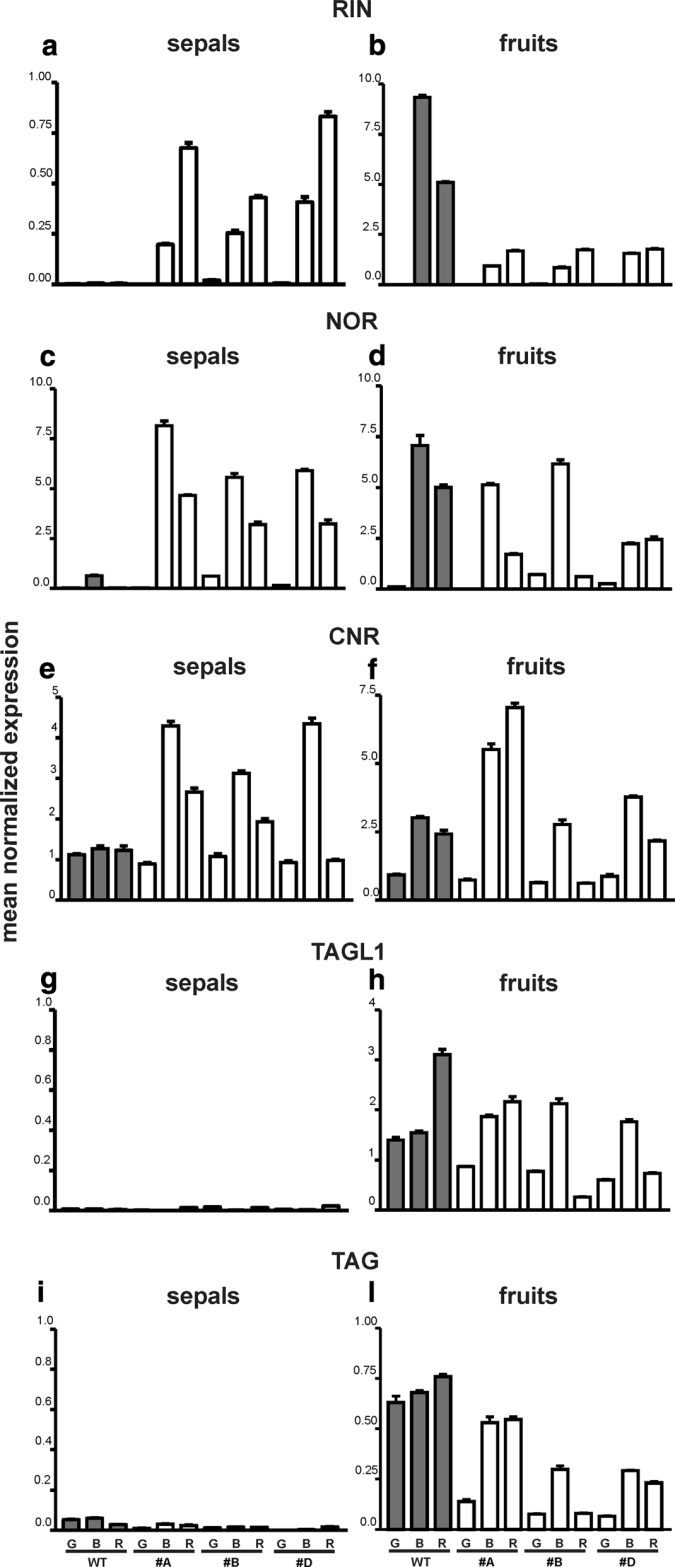
Relative expression profiles of tomato genes coding for ripening-related transcription factors. The analysis was carried out in sepals and fruits of WT and 35S:*GBM5* plants. Grey rectangles represent wild type tissues (WT) while white rectangles represent 35S::*GBM5* tissues from three different lines (#A, #B, #D). G, mature green fruits and their relative sepals; B, breaker fruits and their relative sepals; R, red fruits and their relative petals. The studied genes were *RIN (panels* ***a*** *and* ***b*** *), NOR (panels* ***c*** *and* ***d*** *), CNR (panels* ***e*** *and* ***f*** *), TAGL1 (panels* ***g*** *and* ***h*** *), TAG (panels* ***i*** *and* ***j*** *)*. Values (means of normalized expression) from real-time PCR analyses. Bars: standard deviations

In general, the process of ripening is under the control of various factors, among them a number of different transcription factors [19]. Well known genes coding for transcription factors involved in the control of ripening in tomato fruits are *RIN*, *NOR* and *CNR*, therefore the expression of these genes was analyzed in the transgenic plants. *RIN* showed the expected pattern of expression in wild type fruits, with maximum levels at the breaker stage. Unexpectedly, the level of expression appeared lower in fruits of all the transgenic lines and the maximum transcript amount was always observed in fruits at the red stage (Fig. 8b). In wild type sepals *RIN* transcripts were undetectable while the transgenic sepals showed a pattern of transcript accumulation that had its maximum in the yellowish-orange ones, thus behaving similarly to what observed in the corresponding transgenic fruits (Fig. 8a).

The *NOR* gene appeared similarly expressed in both wild type and transgenic fruits, although in line #D its levels appeared lower compared to the others (Fig. 8d). Some *NOR* transcripts were detectable in the wild type sepals sampled when fruits were at the breaker stage. On the contrary, in the transgenic sepals all the lines showed significant levels of expression with maximum transcript amount in those that accompanied the fruits at the breaker stage (Fig. 8c). In the case of the *CNR* gene comparable expression patterns were observed in both wild type and transgenic fruits, but for those of line #A where the maximum amount was found in the red ones (Fig. 8f). As regards the sepals, some expression was observed in wild type ones where the levels remained constant during development. On the contrary, a ripening-related pattern of *CNR* expression, with maximum levels in those accompanying the fruits at the breaker stage, was shown by the sepals of the three transgenic lines (Fig. 8e).

In order to see whether the over-expression of the Ginkgo *AG* gene might have affected the expression of the two endogenous C-function genes of tomato, also the expression of both *TAG1* (*euAG* lineage) and *TAGL1* (*PLENA* lineage) was analyzed. No changes were observed for *TAGL1* that was expressed in both wild type and transgenic fruits with a pattern that showed an increase after the green stage, i.e., when the fruits had to ripen (Fig. 8g, h). In the case of *TAG1* a somewhat reduced expression was observed in the fruits of two transgenic lines (Fig. 8j). In sepals the expression of both *TAG1* and *TAGL1* was extremely low so it appeared difficult to observe significant changes of expression in both wild type and transgenic ones (Fig. 8i).

## Discussion

In tomato plants ectopically expressing the endogenous C function *TAGL1* gene some degree of homeotic conversion of petals in either stamenoid structures or pollen producing stamens was observed [10], while the ectopic expression of the other C function *TAG1* gene induced also defective anatomy of anthers and ovaries, besides transformation of petals into stamenoid structures [9]. The ectopic expression of the Ginkgo *AGAMOUS* gene in tomato did not cause any evident anomaly at the level of petals, anthers or ovary, all of them having a normal appearance. Moreover, the transgenic fruits grew and ripened regularly, as judged by the analysis of various ripening-related genes. Also the structure of their pericarp did not show any difference compared to that of wild type tomatoes. On the basis of the above results it might seem that the Ginkgo *GBM5* gene has no C function. Yet, this is not true because the above ectopic expression yielded the homeotic conversion of sepals into carpel-like structures, and a similar conversion was also performed by the *TAG1* and *TAGL1* genes [9, 10].

The tomato *TAGL1* and *TAG1* genes belong to different sub-lineages of *AGAMOUS*, hence their functionality was not exactly overlapping and, in fact, some differences were observed in the transformed sepals. In the case of *TAGL1* the sepals were connately adjoined to each other along their entire length as if they tended to form an ovary which, however, was open at the distal end to let the style out; moreover, this ovary-like structure became quite thick and fleshy, and developed a reddish color [10]. The ectopic expression of *TAG1* gave different results that consisted in the formation of fleshy sepals that were connately adjoined only at their proximal end and developed a yellowish-orange color but never a red one [9].

The ectopic expression of the Ginkgo *AG* gene caused an evident anomaly only in whorl 1. The detailed study of the homeotically modified sepals revealed that this gene has a true C function because it was able to cause the sepals to be transformed into carpel-like structures that had many characteristics typical of the fruit pericarp. Carpels can be regarded as specialized leaves folded along the tip-base axis and fused together along their margins to form the ovary, therefore the carpel epidermis corresponds to the abaxial epidermis of a leaf proper. In tomato the carpel-derived pericarp had a region, consisting of a few layers of very small cells, situated right below the epidermis, while the internal region was formed by huge cells with small air spaces among them. In the case of sepals the wild type ones maintained throughout their entire life a more or less leafy structure with only a spongy mesophyll sandwiched between the two epidermises. On the contrary, concomitant with the ripening of the fruits to which they were attached, transgenic sepals changed markedly both their color from green to a yellowish-orange tonality and their structure. In particular, the sepal internal structure resembled that of a tomato pericarp with a few layers of small cells underneath the abaxial epidermis and an internal region with huge roundish cells whose size was comparable to that of the cells situated in the internal pericarp of ripe fruits. Moreover, the “mesophyll” of the transgenic sepals appeared rather compact and with limited and small air spaces, thus mimicking the structure of the fruit pericarp. Interestingly, the absence of any visible anatomical boundary in the region where two transgenic sepals are fused together confirms the idea that those sepals were connately adjoined since the very beginning of their development.

Also the molecular analyses strengthened the idea of the transgenic sepals being fruit-like structures that underwent some ripening, as judged by the expression of some genes used as marker of fruit ripening in tomato. During ripening the wild type tomato berry loses its chlorophyll and accumulates lycopene thus changing its color from green to red. The *PSY1* gene codes for the phytoene synthase exclusively expressed in tomato chromoplasts [24, 25] and, similarly to what occurred in fruits, also the transgenic sepals showed *PSY1* transcript increases in parallel with the change of color, thus showing that they accumulated carotenoids albeit not lycopene because their color never became red.

The process of pericarp loss of firmness represents one of the most relevant aspects of fruit ripening because it makes the fruit pleasant to eat but also quite perishable, and this can cause huge economic losses. It involves the activity of various genes coding for different cell wall degrading enzymes [20, 21]. The expansin gene studied in this work is involved in the destabilization of the cellulose-hemicellulose network and its role was demonstrated by Brummell et al. [26]. The β-galactosidase (*β-GAL4*) gene encodes a protein involved in the degradation of the lateral branches of parietal polysaccharides, thus making the cell wall more accessible to other cell wall dismantling enzymes [20]. The polygalacturonase (*PG*2) gene was used because it is involved in the degradation of pectins and is highly expressed during softening of tomatoes [27, 28]. Interestingly, also these three genes showed a ripening related pattern of expression in the transgenic sepals, thus suggesting that they could undergo at least some limited softening.

The flavor of fruits can be very complex and in ripe tomatoes there are various tens of different compounds that contribute to make up their particular flavor. It has been shown that the enzyme lipoxygenase (*LOXB*) is important for the formation of lipid derived volatiles [22]. However, In tomato there are various *LOXB* genes and one of them appears to be specifically expressed during fruit ripening [23]. But for the ripe sepals of one line, in the transgenic sepals the levels of expression of this gene remained generally low, nevertheless there was some increase during their ripening while the expression of *LOXB* remained undetectable in wild type sepals. Also this finding suggests that the transformed sepals behaved like ripening ectopic fruits.

It is interesting to note that in Ginkgo the ripening of the fleshy fruit-like structures appeared to be a sort of rudimentary process, as judged by the very limited expression of known ripening-related genes [16]. On the contrary, in the fleshy tomato sepals transformed with the Ginkgo *AG* gene various ripening genes appeared to be expressed with a clear ripening-related pattern. The different ripening behavior observed in Ginkgo and in the transgenic tomato sepals suggests that the Ginkgo *AG* gene might play a general regulatory role in the establishment of the fleshy structures that are destined to ripen, while the various pathways involved in the different aspects of ripening (i.e., the ripening syndrome) would be under the control of other regulatory genes in a species-specific manner.

The *RIN*, *NOR*, and *CNR* tomato genes have been shown to control fruit ripening very strictly, such that, when mutated, each of them can cause a block of the ripening process that leaves the mutant fruits at the mature green stage [19]. In wild type tomatoes these genes show an increasing pattern of expression during ripening, with a maximum at the breaker stage. Although in all the transgenic samples *RIN* showed maximum expression levels in those corresponding to the ripe stage instead of the breaker one, also for these genes there was increasing expression in the transgenic sepals as it should occur in fruit proper. Therefore, also regarding the expression of genes involved in the control of the tomato fruit ripening, the fleshy transgenic sepals behaved like fruits.

The ectopic expression of the Ginkgo *AG* gene did not change the expression of either *TAGL1* or *TAG1* in the transgenic sepals, therefore this finding suggests that the observed homeotic conversion of sepals into carpel-like structures had been induced by the Ginkgo *AG* gene. Surprisingly, the level of expression of the *TAG1* gene was found to be lower in the fruits of two transgenic lines, thus suggesting the occurrence of some co-suppression. However, the fruits produced by those two lines had a normal appearance and ripened regularly, therefore the observed lower transcript amount was apparently still enough for a normal development of those fruits.

As already mentioned, in core eudicot Angiosperms the *AGAMOUS* lineage is divided into two sub-lineages [6]. Actually, a duplication of *AGAMOUS* was evidenced later on in basal eudicots [29], and also in Magnoliids and Nymphaeales [30]. However, in a comprehensive phylogenetic tree of AG proteins the latter duplications did not group with either the euAG or the PLE sub-groups, therefore the position in a phylogenetic tree might be useless in order to obtain suggestions about the possible physiological roles played by the duplicate genes in basal Angiosperms, although in the case of *Magnolia grandiflora* the different expression patterns of the two AG genes suggested that one of them could possibly play a role in the formation of the red and fleshy sarcotesta that surrounds the seeds of Magnolia [30]. In the case of Gymnosperms it would be even more difficult to make comparisons with the roles played by the euAG and PLE proteins of core eudicots because all the Gymnosperm AG proteins normally group together in phylogenetic trees where also Angiosperm AG proteins are considered (see Fig. 3 in [10]). Therefore, a phylogenetic tree comprising sequences of the euAG and PLE groups would be useless also for the Ginkgo AG sequence.

## Conclusion

The functional analysis carried out in this work showed that the Ginkgo *AG* gene was able to cause the homeotic transformation of tomato sepals into fruit-like structures that could undergo some ripening, albeit the latter process was not complete since the “ripe” sepals were mostly yellowish and not too thick. This result indicates that, from a functional point of view, the Ginkgo *AG* gene is more similar to the *euAG TAG1* gene rather than to the *PLE TAGL1* gene, and suggests that the *euAG* form of *AGAMOUS* would be more representative of the ancestral *AG* gene compared to the *PLE* form. Furthermore, we showed that the Ginkgo *GBM5* gene has a genuine C function, and that it could be involved in the formation of the Ginkgo fleshy fruit-like structure surrounding the seed, thus confirming the idea that the suite of MADS-box genes involved in the development of the fleshy fruit habit was already active in Gymnosperms as ancient as the Ginkgoales [16].

## Methods

### Plant material

*Ginkgo biloba* tissues used to clone the *GBM5* cDNA and to study its expression profile were obtained from plants growing at the Botanical Gardens of Padua. Tomato seeds (*Lycopersicon esculentum* cv. Florida Petite) were purchased at the Tomato Growers Supply Company, Fort Myers, FL, USA. Tomato plants were grown under standard conditions at 25 °C and a 16-h photoperiod in a greenhouse at the Department of Biology, University of Padua. No authorization was needed for growing the tomato plants in the above greenhouse. Fruits were harvested at different stages of development: mature green (G), breaker (B) and red (R). Sepals from fruits at each of the above stages of development were also collected. All tissue samples were frozen and stored at −80 °C for further use.

### Transformation of tomato plants

The Ginkgo *GBM5* (Accession number: AY114304) cDNA was isolated from young ovules using specific primers (FOR: ATGGGCCGTGGGAAGATTGAGAT and REV: ATCCCGCCCATAAACTTCATCCA). The identity of the obtained cDNA was ascertained by sequencing. The DNA sequencing was performed by BMR Genomics, Padua, Italy. Sequence manipulations, analyses, and alignments were performed using the LASERGENE software package (DNASTAR).

The complete *GBM5* cDNA was cloned into the pBin-AR vector [31] and the resulting binary plasmid was inserted into *Agrobacterium tumefaciens* cells (strain LBA4404) that were used to transform tomato seedlings according to [32]. Kanamycin-resistant plants were confirmed for the presence of the transgene by means of PCR.

### RNA extraction and gene expression analysis

Total RNA was extracted from different tissues according to [33]. RNA yield and purity were checked by means of ultraviolet (UV) absorption spectra and the integrity was ascertained by electrophoresis in agarose gel (data not shown).

The RNA samples obtained from different tissues were converted to cDNA by means of the High-Capacity cDNA Archive Kit (Applied Biosystems), using random hexamers as primers. 3 μg of total RNA, pre-treated with 2 U of DNase I (Promega), were used as starting template.

The gene expression analysis was performed by standard real-time PCR. Primer sequences for the selected genes are listed in Additional file 2. The normalization was performed using the ACTIN cDNA for tomato and the internal transcribed spacer (ITS) of the ribosomal RNA sequences for ginkgo. PCR was carried out with the Gene Amp 7500 Sequence Detection System (Applied Biosystems). The obtained C_T_ values were analyzed by means of the Q-gene software by averaging three independently calculated normalized expression values for each sample. Expression values are given as the mean of the normalized expression values of the triplicates, calculated according to equation 2 of the Q-gene software [34].

### Light microscopy analysis

Sepals and fruits from wild type and transgenic plants were cut into little pieces that were subsequently fixed in 3 % glutaraldehyde in 0.1 M cacodylate buffer (pH 6.9) for 24 h and post-fixed for 2 h in 1 % osmium tetroxide in 0.1 M cacodylate buffer, dehydrated in a graded ethanol series and then embedded in araldite resin. Thin sections (1 μm), obtained with a Reichert-Jung ultramicrotome, were stained with 1 % toluidine blue for light microscopy.

By using the imaging software Leica Application Suite associated to the Leica DM 5000 B automated upright microscope, quantitative analyses were performed. In particular it was measured the cell area of 100 cells/each sample in1 μm thick sections obtained from three different control and transgenic fruits or sepals, respectively. The obtained values were expressed as the mean ± SE of 3 independent replicates for each sample, using the Student’s *t*-test to analyze the differences between the control and transgenic groups. Statistical significance was set at P < 0.05.

## Additional files

Additional file 1:
**Examples of fruits of #B and #D transgenic lines.**
Additional file 2:
**List of primers used for the expression analysis.**
