# Supplementary figures and images for: Characterization of an AGAMOUS gene expressed throughout development of the fleshy fruit-like structure produced by Ginkgo biloba around its seeds
Source: BMC Evol Biol. 2015 Jul 16;15:139. doi: 10.1186/s12862-015-0418-x (PMC4502469; doi:10.1186/s12862-015-0418-x)

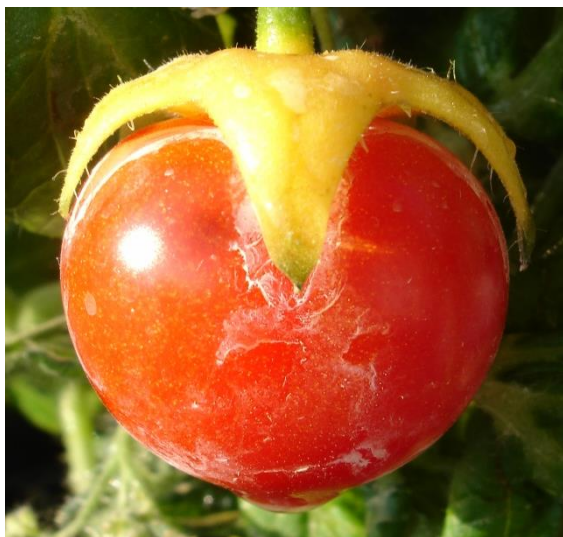

#B

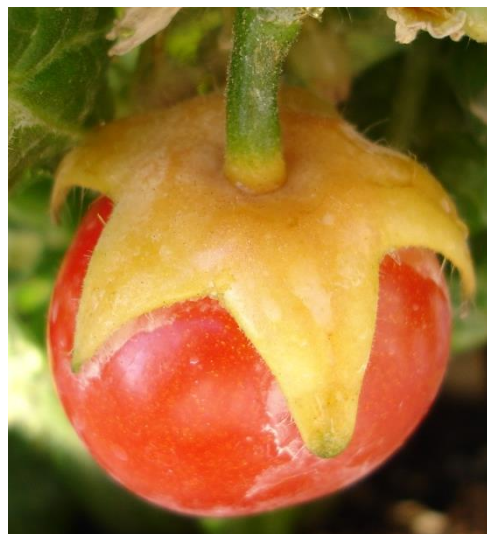

#D

Supplement: Additional file 1: — Examples of fruits of #B and #D transgenic lines. [file 12862_2015_418_MOESM1_ESM.pdf]
