# Supplementary material for: Characterization of an AGAMOUS gene expressed throughout development of the fleshy fruit-like structure produced by Ginkgo biloba around its seeds
Source: BMC Evol Biol. 2015 Jul 16;15:139. doi: 10.1186/s12862-015-0418-x (PMC4502469; doi:10.1186/s12862-015-0418-x)
Supplement: Additional file 2: — List of primers used for the expression analysis. [file 12862_2015_418_MOESM2_ESM.pdf]

GENE NAME	OLIGO FOR NAME	SEQUENCE OF OLIGO FOR 5'-3'	OLIGO REV NAME	SEQUENCE OF OLIGO REV 5'-3'	GeneBank Acc. N.
Ginkgo biloba ITS	GbITS For	GCGGTCGGGAAGGATGTGC	GbITS Rev	GCCGAGGGGAAATGCGAGAAG	EU643829.1
Ginkgo biloba AG	GbAG For	TGAATGTGAAAGCAGCCAAAAT	GbAG Rev	ATCCCGCCCATAAACTTCATC	AY114304
Solanum lycopersicon ACT	TACT For	AGGCACCCCTATATCCCAAG	TACT Rev	AAGCACAGCCTGGATAGCAAC	AB199316
Solanum lycopersicon EXP1	TEXP1 For	ATTACGCGTTGCCAAATGACA	TEXP1 Rev	AGCGCGGTACTGAGCAAGTT	U82123
Solanum lycopersicon β-GAL 4	TBG4 For	CTACTGCGAAGGGTTCCGTC	TBG4 Rev	TTTGAGGAATTGGACCACCG	AF020390
Solanum lycopersicon LOXB	LOXB For	CAAACACAAAAGCCTATGCCTCAA	LOXB Rev	TGCCAGATAGAACCTTCAACACCT	XM_010325709.1
Solanum lycopersicon PG2	PG2 For	GAGGAACTATCAATGGCAATGGA	PG2 Rev	CCAGAAGGTTAAGGCCGTTG	X04583
Solanum lycopersicon PSY1	PSY1 For	CGTTTTTTTGATTCATCGAGGC	PSY1 Rev	CGTACAGAAAATTTCCGTCCATT	M84744
Solanum lycopersicon CNR	CNR For	AACAAATGGGAAGGGAAGAGAAGC	CNR Rev	GCACTGATCGACCTGGCAAGAA	DQ672601
Solanum lycopersicon TAGL1	TAGL1 For	TCAGCCAAATTACGAAGATGC	TAGL1 Rev	AAGCTGGAGAGGAGTTTGGTCA	AY098735
Solanum lycopersicon TAG1	TAG1 For	TGCCAGGGAGTTCATCAAA	TAG1 Rev	GGTTGGTCTTGTCTAGGGTAATG	AY098733
Solanum lycopersicon NOR	NOR For	ACGATGCATGGAGGTTTGTATTG	NOR Rev	TTAAGTCCATCGTCCTCGTTGTTC	AY573802
Solanum lycopersicon RIN	RIN For	AAACATCATGGCATTGTGGTGAGC	RIN Rev	ATGGTGCTGCATTTTCGGGTTGTA	AF448522
